# “Thinking about the future, what’s gonna happen?”—How young people in Sweden who neither work nor study perceive life experiences in relation to health and well-being

**DOI:** 10.1080/17482631.2017.1422662

**Published:** 2018-01-16

**Authors:** Ulrika Lögdberg, Bo Nilsson, Catrine Kostenius

**Affiliations:** ^a^ Department of Health Sciences, Luleå University of Technology, Luleå, Sweden; ^b^ Department of Culture and Media Studies, Umeå University, Umeå, Sweden

**Keywords:** Health, health promotion, NEET, social exclusion, inclusion, qualitative study, well-being, young people

## Abstract

**Purpose:** The aim of this study was to explore how young people in Sweden who neither work nor study perceive life experiences in relation to health and well-being.

**Methods:** A task-based interview technique was used and data was analysed with qualitative content analysis. Interviews were conducted with 16 participants aged 16-20 who were unemployed and not eligible for upper secondary school, or who had dropped out of school.

**Results:** Three themes emerged from the analysis illustrating how the young people perceive their life experiences in relation to health and well-being: *Struggling with hardships in the absence of caring connections, Feeling good when closely connected to others*, and *Being forced to question what has been taken for granted*. Each theme consists of 2-3 subthemes.

**Conclusion:** Based on the young people's narrated experiences health can be understood as: something that is created in relation to others and in relation to the social and cultural context; as something dynamic and changeable; as the ability to adapt and respond to challenges; and finally as something existing on a collective as well as an individual level. Implications for school, social services and health promotion initiatives are discussed, with an emphasis on working with young people.

## Introduction

This article focuses on health experiences of young people in Sweden who neither work nor study. Health in Swedish youth has become increasingly unequal (Public Health Agency of Sweden, 2013), and Povlsen, Borup, and Fosse (2011) stress the need for a stronger focus on health equity within health promotion in the Nordic context. This is in line with the World Health Organization (WHO) who lists health equity as one of the goals of health promotion (WHO, 1986). According to research and governmental reports, young people in Sweden aged 16–24 have had worse health developments than other age groups in recent decades (Hagquist, 2010, 2011; National Board of Health, 2009; Safipour, Higginbottomb, Mesfin, & Emamid, 2012). For example, mental health issues and problems have increased among older adolescents (Hagquist, 2010). Even if the conditions for health among children and adolescents have improved over time, the difference between groups has increased (Public Health Agency of Sweden, 2013). Over the past 10 years, approximately 17,000 young people in Sweden between 16 and 19 years of age have neither been in school nor had employment (SOU, 2013).

From an international perspective, in the countries participating in the Organisation for Economic Co-operation and Development (OECD) in 2010, around 17 million young people age 15–24 were considered a young person Not in Employment, Education, or Training (NEET; Nordenmark, Gillander Gådin, Selander, Sjödin, & Sellström, 2015). Education and employment are the most important social determinants of health (Marmot, 2005). A lack of education may have consequences for an individual’s future opportunities to establish themselves in the labour market (SOU, 2013). Furthermore, research shows that youth unemployment contributes to health problems later in life (Hammarstrom & Janlert, 2002). According to Swedish and international reports, leaving school early can lead to social exclusion and failure to develop a sense of the future (Pålsson, 2012; Stengård & Appelqvist-Schmidlechner, 2010). Young people outside the school system and labour market are a heterogeneous group who face common and individual challenges connected to future health and well-being; they often find themselves in vulnerable situations (SOU, 2013).

However, the NEET terminology has been criticized for not recognizing differences within the group: young people who are unemployed for a longer or shorter period of time, young people caring for their children, temporarily sick or long-term disabled, or those just taking a short break from work or education for travel or other interests (Furlong, 2006). Other reasons for being included in the NEET group in the Swedish context are social problems and school failure, mental illness or having recently arrived to Sweden as young migrants (SOU, 2013). Hence, the rationale for being out of work or school is not clear and motivates this work.

To increase young people’s health, the United Nations Children’s Emergency Fund (UNICEF, 2011) and the WHO (Currie, 2012) stress the need to change adolescence to a time of opportunity instead of one of risk. According to Randell, Jerdén, Öhman, and Flacking (2016), there is a lack of salutogenic research focusing on the root causes of health. The central issue here is how to define health. The World Health Organization defines health as “… a state of complete physical, mental and social well-being and not merely the absence of disease or infirmity” (WHO, 1946, p. 100). Well-being can be viewed as a person’s subjective experience of health (WHO, 1986). Huber et al. (2011) add to the discussion of defining health and viewing it from an action perspective investigating people’s sense of ability to adapt and respond to challenges. In addition, Wilkinson and Marmot (2003) point to health as something that is created and made possible in a social and cultural context and not as a purely biological condition. This is in line with the health promotion perspective where health is viewed from a holistic point of view, e.g., Antonovsky (1987). He is the founder of the salutogenic model, an approach focusing on health promotion, and describing health as the ability to comprehend, manage and experience meaningfulness in different life situations. In the Ottawa Charter, health promotion is defined as “the process of enabling people to increase control over and to improve their health” (WHO, 1986, p. 1).

Despite the great attention young people who neither work nor study has received in the Swedish and international discourse, more research about this group is needed to support their health development (Nardi et al., 2015; Niknami & Schröder, 2014; Nordenmark et al., 2015). In addition to survey-based studies on the NEET population focusing on differences within the group of NEETs (Nordenmark et al., 2015) or between the NEET and their EET peers (in education, employment or training; Nardi et al., 2015), there is according to Woodgate and Leach (2010) a need for investigation that deal with young people’s perspectives on what creates and affects health. In addition, Larsson, Johansson Sundler, and Ekebergh (2012) argue that health experiences must be understood in relation to young people’s life experiences and their own conceptualizing of these experiences. Some studies deal with young people’s health perceptions in a Swedish context (Einberg, Lidell, & Clausson, 2015; Larsson et al., 2012; Randell et al., 2016). Even though these studies focus mainly on health experiences, the participants were recruited exclusively in school. With this in mind, it is important to study how young people, both inside and outside the school environment in Sweden, experience health.

In sum, young people who are not employed or in school are considered a vulnerable group with a higher risk of future illness relative to their peers who are employed or students (Nordenmark et al., 2015). To achieve health equity among youth, more research is needed to understand how young people’s health and well-being can be improved, and how they can fulfil their potentials. In the Swedish context and from an international perspective, there is limited research concerning this group of youth and their own experiences in relation to health. To empower young people—and to improve their health and well-being—their perspectives should be utilized. This can be accomplished with qualitative research based on participants’ views (Sofaer, 1999). Therefore, the aim of this study was to explore how young people in Sweden who neither work nor study perceive life experiences in relation to health and well-being.

## Method

A qualitative design was used comprising qualitative interviews and a qualitative content analysis with an inductive approach. The study was based on an assumption that knowledge is created through interactions between researchers and participants (Kvale & Brinkmann, 2009). Therefore, a task-based interview technique inspired by Conolly (2008) was used because it can provide insights into an individual’s life and values while at the same time empowering the participants. In the interviews, participants were invited to describe their life in a broad sense and to reflect on situations related to their health and well-being.

### Participants

This study included 16 young people aged 16–20 who were unemployed at the time of interview were not eligible for upper secondary school, or who had dropped out of school. To increase variation of experiences, participants were recruited through three different national or community-based initiatives in Sweden. These initiatives were directed towards young people who were not attending a national program within a secondary school or who were unemployed.

The first initiative was directed at newly arrived youths. They were coming to Sweden for different reasons such as fleeing from war alone or with family, moving with one or two parents who either started to work in Sweden, or who had a partner in Sweden. The participants came from Asia, Africa and Europe and had different school backgrounds: some had never been in school while others had been in school for 16 years in their home countries. The second initiative involved young people (primarily up to age 18) who needed support and help to create and formulate their goals in life and to explore ways of reaching them; a number of the young people in this group had special needs. The initiative was as an alternative to upper secondary school offered to young people who had experienced challenges in the compulsory schooling or secondary school or was not eligible for upper secondary school. The third initiative was a 6-week course for young people who were unemployed job-seeker, missing grades from the compulsory schooling or had incomplete grades from secondary school. All participants in the second and third initiatives were born in Sweden.

The social backgrounds of the participants differed; our sample included both males and females who were born in Sweden or had immigrated to Sweden the past couple years. The socioeconomic status of young people is often defined by their parents’ position in the labour market, education status, or income (Currie, 2012), however our participant’s descriptions of the socioeconomic background of their parents varied from unemployed, low-income job, to high-income job. Several participants did not live with their parents and had no contact with them. The staff at these programs received written and oral information about the study and thereafter invited the young people to participate in the study.

### Data collection

The interviews were conducted by the first author in the young peoples’ everyday environment (i.e., locations at the initiatives where they spent their days) in order for participants to feel safe (Morrow & Richards, 1996). Each participant was interviewed individually and the interviews lasted between 22 and 77 minutes. The average interview lasted 47 minutes. The participants were asked what language they would like to communicate in if Swedish were not their mother tongue. All of them were comfortable speaking Swedish and therefore all interviews were held in Swedish. When interviewing children and young people, it is important to provide true opportunities for them to say no, especially if parents or another significant adult has already agreed to the young person’s participation (Coyne, 2010). In this case the staff at the initiatives where the young people were recruited was part of saying yes to the study. Therefore, the first author was extra sensitive to signals of (dis)interest in continuing their participation (Mahon, Glendinning, Clarke, & Craig, 1996).

The interview questions focused on the everyday experiences of the young person. Participants were encouraged to narrate their upbringing and describe what their daily life looks like today. The interviewer followed up on their stories and asked participants to describe significant life events and reflect on these events in relation to health and well-being. As a stimulus to promote conversation, all participants were invited to perform task-based activities, which included completing a timeline with two questions: Events in life that have made me feel well (past) and future events that would make me feel well (future). Participants’ written and oral reflections were followed up with questions like “How did this make you feel?” and “Can you tell me more about…?” to enrich the data, in accordance with Rutberg, Kostenius, and Öhrling (2013). All participants chose to interact with the timeline exercise in different ways. Most drew a timeline and highlighted events, some drew a picture, and a few just responded to the questions. Finally, all participants were asked to finish the following sentences inspired by Conolly (2008): When I´m with my friends we spend our time… When I’m at home, I feel … When I finish this program … When I think of the future … I feel important when … It is meaningful to me…

### Data analysis

The analyzing process started with the first author listening to the recorded interviews and transcribing them verbatim. All authors read the text as a whole to obtain an initial sense of the data, which was then followed by a discussion between the authors about questions as: What are the young people describing? What feelings are the young people expressing to let us know what the experiences are like for them? What are the young people telling us about their lives? How can we understand their described feelings and experiences in relation to health and well-being (Kostenius, 2008)? Each interview was coded with the support of the program NVivo by the first author. This step was characterized by an analysis that was close to the text and dealt with the more “visible, obvious components,” referred to as manifest content (Graneheim & Lundman, 2004, p. 106). The three authors discussed the codes and preliminary themes on several occasions. The next step of the analysis was characterized by a more latent analysis and dealt with the “relationship aspect and … interpretation of the underlying meaning of the text” (Graneheim & Lundman, 2004, p. 106). Representative and illustrative quotations from the data material were chosen to exemplify the themes, not only giving voice to the young people but according to Polit and Beck (2004) enhances the credibility of the study.

### Ethical considerations

Creating data in conjunction with young people requires a reflective ethical approach throughout the whole research process—especially for this group of young people who can be considered a vulnerable group since they are not employed or participating in the education system (Valentine, Butler, & Skelton, 2005). The study was conducted with the approval of the Research Ethics Committee in Umeå, Sweden (Dnr 2013–462-31Ö). Before agreeing to participate in the study, all participants received written and oral information in Swedish about the aim of the study and they had the opportunity to ask questions to clarify potential uncertainties about the study and their participation.

Because all of the participants were over age of 15, informed consent was only collected by the participants and not from their parents or another significant adult in accordance with the ethical vetting law in Sweden (SFS, 2003:460, §18). All participants received written information about the study that they were encouraged to bring home. They were also notified that their participation was voluntary, that they could cancel their participation without explanation at any time, and that they would not be identified in the written and published text. To give the participants the opportunity to reflect on their participation and to make sure that the data captured the young people’s perspective, the interviews ended with questions probing if the participants wanted to add, withdraw, or highlight any issue (Sand, Emaus, & Lian, 2015). Interviews can be emotional experiences, and thus they were offered the contact information to a social worker in case they wanted to talk further about anything that surfaced during the interview.

## Results

Three themes and seven subthemes emerge from the analysis (Table I). The results illustrate how the young people perceive their life experiences in relation to health and well-being.

**Table I.** Overview of the findings in themes and subthemes.

Theme	Subthemes
Struggling with hardships in the absence of caring connections	*Being in the middle of war, loss, and displacement* *Having to deal with drugs and violence* *Feeling betrayed*
Feeling good when closely connected to others	*Feeling closely connected and supported* *Doing something meaningful and being recognized*
Being forced to question what has been taken for granted	*Finding oneself in a new life situation* *Looking back or fighting for the future*

### Struggling with hardships in the absence of caring connections

The first theme represents the young people’s experiences of hardship in life through the following subthemes: *being in the middle of war, loss, and displacement; having to deal with drugs and violence*; and *feeling betrayed.*

#### Being in the middle of war, loss, and displacement

For the young people, experiences of war signified being forced to leave home and being apart from family. They described situations where they waited for and worried about whether or not family members would return from war, not knowing if they would. They also described the fear of losing these family members. One young person said,

One day they started a war and then the whole family disappeared, they ran back and forth and then my brother moved from Somalia to Sweden…we think he might have died, we have no idea. (20-year old male, P11)

As illustrated in this example, the young people’s stories of war reflected the uncertainty they felt about their life situation in that moment. The uncertainty encompassed the well-being of their families and loved ones as well as their own life circumstances. Their own life circumstances included, for example, fleeing their home country not knowing where they would end up and how the future looked for them. They also described experiences of leaving family behind and losing significant others. Besides the context of war, another reason for leaving home was related to tough family situations. The young people described moving around Sweden with parents who sought employment or moving between divorced parents. According to the young people, these situations resulted in feelings of not being welcomed and not feeling at home anywhere. One young person said,

I cannot say I really feel at home anywhere … if it feel like they don’t want me there it’s not like I want to be there… if I’m with my dad then Mom starts calling and picks a fight. Dad does not want any trouble so then he shoves me away back to Mom, Grandma is like doing the same, shoving me back to Mom … Everyone wants to push me to my home because they do not want to take responsibility, and I would not be where I do not feel comfortable. (17-year old female, P5)

They also described leaving home because of complicated relationships with their parents, being placed in foster care, or falling into the care of the government due to drug abuse and domestic violence. Regardless of their reasons for leaving home, the young people felt forced to move because their family or the government made the decision.

#### Having to deal with drugs and violence

Having to deal with drugs and violence was another subtheme that emerged from the descriptions of their upbringing and everyday lives. Drugs such as alcohol and cannabis were described as a part of family life (when coming from a family with drug abuse) or as a natural part of being an adolescent. Connected to family life, they talked about drugs as a reason for hardship in their childhood and at the same time as a survival strategy when feeling down, stressed, or sad, and as a way to escape from bad memories and experiences. One young person explained his relationship to drugs; his family had introduced him to a life with drugs.

Well, eh, my childhood has been kind of yea pretty tough…when I had a family I did drugs, you know drug abuse…eh, you know I saw drugs and those things when I was pretty damn young … I thought drugs were cool and like lot of money… yea the way I acted I learned from my dad you know and my other brother… they were my role models I wanted to be like them. (20-year old male, P1)

Drugs were also described in relation to violence. They shared stories about getting hit by friends when drinking for the first time as well as parents coming home drunk and hitting other family members. They also shared stories where they were the one perpetrating violence against others. However, violence was not just present in the young people’s stories about drugs; violent behaviour was also described in other societal contexts such as in school, within their family, and in relation to governmental care. The young people described experiences of school as an unsecured context where fights with other classmates, harassment from teachers, and bulling took place. They expressed feelings of psychological violence and mistreatment. As one young person said,

… my teacher was harassing me too … He got on my case all the time when someone else in the class talked and told *me* to be quiet—not anyone else … He picked on me because I have ADD [Attention Deficit Hyperactivity Disorder] and problems with concentration… (18-year old male, P8)

They described encounters with physical violence within governmental services and within families, resulting in feelings of powerlessness. One young person said,

One time when I was not feeling so well my mom and dad were fighting … We didn’t, I didn’t have a good life… I heard they said something like, you know they can hit their wives in Africa… that’s how it was, my dad hit my mom and I heard it and you know I couldn’t sleep ‘cause I couldn’t do anything. (19-year old male, P14)

Drugs and violence were sometimes related in the young people’s stories, but this was not true in all cases. Both drugs and violence were described as phenomena existing on an institutional level (in connection to governmental care or within school) and on a group level (the family or groups of friends).

#### Feeling betrayed

Another aspect of hardship the young people described were situations where they reached for or longed for support and caring connections yet felt betrayed. They expressed disappointment that family members were not there for them when they needed them, that family members were not there at all, or that they were only there in crises. A couple young people described it this way:

Well you know it didn’t get any better, we were just running out of tears or something, it was only to go to bed and hope she’d come back in the morning (17-year old female, P9)

…she is there for me when I feel really bad, but then it’s like she lets go of me when I feel better. (17-year old female, P5)

They described turning outside the family for support and being given demands they could not fulfil. For example, one young person described wanting help from social services to—among other things—quit drugs, however she was refused help if she was involved in drugs. When describing betrayal in relation to lack of support, they described feelings of not being listened to and not being fully understood. They described how meetings within governmental services left them feeling powerless and distrustful. One young person explained,

I don’t know. He [The social worker] wanted me to live there but I didn’t know anything about it. Nothing at all. You know he said nothing about me moving…I found it out in a meeting at the social care office. I was going to be placed in a foster home …that was damn tough. First I thought: Hell, I don’t want to live with someone else’s family, I have my own family. Then I—but I left, you know ran away … But then, yea as usual, the cops got me after a while and then, well, then I said I move, you know I said that I can move there. (20-year old male, P1)

This example illustrates, the young people had experiences where others made decisions about their future without involving them. They also talked about the betrayal of close friends and described how they had to handle the sadness and disappointments by becoming tough. One young person said “I’ve learned to take care of myself. We’re better off on our own, I think” (17-year old female, P9). Altogether, the theme *struggling with hardships in the absence of caring connections* illustrates situations in the young people’s life where their well-being was limited due to feelings of lack of control, disappointment, and lack of trust in others as well as feelings of uncertainty and worry.

### Feeling good when closely connected to others

The second theme, *feeling good when closely connected to others*, communicates the young people’s experiences of feeling good in life. It does so through two subthemes: 1) *feeling closely connected and supported*, and 2) *doing something meaningful and being recognized.*

#### Feeling closely connected and supported

According to the young people in this study, feeling closely connected to others and being supported was central to their ability to feel good in life. The subtheme illustrates that relationships with their families, friends, and others in their vicinity were a source of well-being. Through the support of others, the young people felt empowered to make a positive life change or to continue doing something that made them feel good. They highlighted, for example, communities such as sport situations, and described how the support of a coach and team members helped them during hard times. Furthermore, they described how people outside the family could provide support and help bring hope for the future. According to the young people, a number of non-family members had become significant in their life. Examples included friend’s parents, parents’ new partners, and social workers. The young people described how these significant figures introduced them to new places and helped them discover hobbies, bringing much pleasure and joy to life. One young person said,

… my other dad, he’s actually like my dad, my best friend and mentor. And he introduced me to fishing, wilderness, nature, and even jobs. My mom helped me a lot with school and like that and my real dad helped me with practical things, driving a car and getting a taste of real life or how do you say. My pretend dad, yea you know, he is the most important. (18-year old male, P8)

The interviewees’ also emphasized relationships outside their own families, because they considered such relationships to be a gateway to a better life. For example, living with foster parents gave some young people a chance to experience a life where they felt like part of a family and could live in an environment without drugs and violence. One young person said,

…it was there I like got to see that there’s another life, you know…it was a good family…they were a hockey family and the dad was a coach and the sons were playing hockey…so I started working out again…it felt damn good. It was like a good family. They really took me in, as if I was a family member. (20-year old male, P1)

Outside of relationships with adults who could offer support and guidance in life, friendship with peers were central to the young people in order to feel good. They also discussed temporary relationships and short encounters that had meant a lot to, and offered them support especially during hard times. They described interactions at social services and in school, where they met people who really listened to them and tried to improve their situation. This support was especially appreciated in situations when they felt that nobody else paid attention to them or cared. The common thread in these experiences is that the young people had a sense of being listened to and being understood. They described feeling supported in situations where they were included and respected, where they felt that they were treated as an individual with a unique life story instead of being given a pre-determined, fixed solution. One young person explained,

…it was like one of my social workers… she was sensible. She really tried that, how do you say it, you know she really worked hard to try to make it as good as possible for me. The other ones they like no you must be locked up, like that… (20-year old male, P1)

The young people described feeling supported as having someone recognize you on an everyday basis and having someone to turn to in times of need, for example in crisis situations.

#### Doing something meaningful and being recognized

According to the young people, positive memories and good experiences of doing something meaningful in their childhood made them feel good in their current life. They described the good life through a sense of belonging with others and being connected to the life in the village back home, for example when playing soccer with friends or building huts. According to the young people, being able to run over to friend’s houses in the neighbourhood, playing all day long, and constantly being with friends and family created a sense of ease. Besides being at home and being with family, the young people highlighted contexts such as school, sports, daily activities, and “being on the net” as meaningful spaces for feeling well in life. Activities that made them feel good meant having something meaningful to do every day, which gave them a sense of purpose. They compared times when sat at home not doing anything with the feeling of doing something meaningful. According to the young people, this could occur in different contexts, for example in training for secondary school or in Alcoholics Anonymous meetings, where they felt like they were on their way to something better.

Involvement in sports such as playing soccer, hockey, and volleyball was described as meaningful. They described how playing sports when growing up made them feel important—in this context, they were someone others counted on and looked up to. Sports also functioned as a form of stress management, as it gave them a break from their current problems and allowed them to forget about the past. Besides organized sport activities, hobbies that made life meaningful and enjoyable included fishing, snowboarding, and being on the internet playing games and talking to others. The internet facilitated relationships that could be difficult to create in ordinary life. One young person explained,

If I sit online, then I might sit and talk for a whole day with just anyone I only met a week ago. Like there’s nothing strange about it. But otherwise it’s just kind of sitting here talking with friends, like so. It is not likely that someone start a conversation with just anyone. I don’t go up to someone and start saying—“how is your life” … like that. I’m not like that. (16-year old male, P2)

Internet activities that could be performed with siblings and friends, such as playing video games or using Photoshop, were also described as meaningful. Furthermore, these activities allowed them to be acknowledged for their skills, for example when advancing in games and getting likes for posting pictures. Furthermore, having a good relationship with nature, animals, or God made them peaceful. One young person said “When I feel good … I pray … ‘cause I don’t know how but I can say it… I’m on another level … I become myself” (19-year old male, P14). To summarize, the theme *feeling good when closely connected to others* communicates the importance of social relationships and network for the young people’s experiences of health. The theme illuminates how being supported and recognized by others was meaningful for their well-being. Furthermore, it shows the significance of meaningfulness for a sense of well-being.

### Being forced to question what has been taken for granted

The third theme, *being forced to question what has been taken for granted*, reflects experiences that led to a reorientation in their lives. This theme was discussed through the subthemes *finding oneself in a new life situation* and *looking back or fighting for the future*.

#### Finding oneself in a new life situation

The narratives marked events when life took a new turn, forcing them to look at life or themselves in a new way. Three different life-changing experiences were identified: leaving home due to war; experiencing a new school context; and quit taking drugs. Leaving home because of war was one example of an experience that they saw as the end of one life and the beginning of another. One young person said,

We were all three going to come together but my brother, he did not dare to leave my siblings and parents back home in Afghanistan. And he sent me further on and he said he would come behind me, after me, and when I came to Greece he said that he cannot come and I’ll go ahead and create a new future. So since then I’ve also lost contact with them. (17-year old male, P4)

Reflecting the life situation they were in, the young people framed the experience of coming to Sweden from other countries in different ways. For some, losing contact with family forced them to start over on their own. Managing life on their own, being separated from family, and not knowing what happened to loved ones caused great distress and feelings of loneliness. They described difficulty sleeping at night due to thinking about their families and what their life was like growing up. One young person said, “It doesn’t feel okay cause I lost contact with my uncle…and being alone, all alone in the world. It’s tough” (18-year old male, P10). Others described leaving their home country and coming to Sweden as a chance to reunite with family and start life together in a new context. Despite this reunion, some participants expressed feeling lonely in their new life situation. One young person said, “It’s only I who moved to Sweden and I think we are the only ones who are from my country in Sweden (18-year old male, P6)”. They also described fear of racism and feeling unsafe in public areas. Living a new life in Sweden often meant facing a new school system or being in school for the first time. This was described as both a challenge and an opportunity. One young person said,

It’s hard to learn two languages at the same time if you never have gone to school. If you are writing in my language it’s hard … It’s a bit tough to sit in school and do nothing… not moving. Sport is good … I like to work hard… (18-year old male, P10)

Experiencing a new school context was described in relation to coming from other countries but also changing schools within Sweden. Participants described leaving school because of bulling and periods of feeling bad as another example of a life-changing experience where they reached a point where they could not go on and had to make a change. Several described coming to a school where help was available after leaving a school because bullying, feeling unsafe, and not getting the right help. One young person described the following situation:

I didn’t get much help… it was quite tough … yea you know I like much more this school … If I can choose what I like the most … the schooldays are of course much shorter here because otherwise I’d be too tired to go on … and then it’s the help I get there. And also, I feel much safer here (17-year old male, P15).

When the interviewees described experiences of leaving school, they expressed feelings of loneliness and they also tried to understand and explain why they had to leave. Sometimes participants blamed themselves, as illustrated in this quote:

They were like really nice and so. But I didn’t get really close to anyone and you know. They were of course very nice and it was a great class. But, nah. So it can be … Well of course they were funny and kind and so but you felt like you were not really a part of that … Well also it gets like you ‘re getting used to sit there alone finally a little bit (20-year old female, P3)

Quitting drugs was a third example of a life-changing experience; in this one, the decision came from hitting bottom, where they just had to make a change. The decision to quit drugs affected other areas in their life and led to several changes. These changes were experienced as both good (e.g., finding new friendships and situations that helped them to find the power to make a positive change) and bad (e.g., leaving home and ending up with nowhere to live). Finding out that they were not alone in their life situation was described as a positive aspect of this life change, whereas quitting drugs also meant leaving family and friends behind. Experiencing the feeling of not being alone in the situation and making a change together with others were described as helpful for making positive changes in life. Altogether, these life-changing experiences signified finding themselves in a new environment with new responsibilities. Furthermore, finding oneself in a new life situation encouraged reflection on their independence and their ability to manage life on their own. They expressed two stances on independence: the will to be able to manage life on their own and yet the realization they needed the support of others. This concerned both practical matters such as handling budgeting and house hold chores, decisions affecting their health such as what to eat and when to sleep, and emotional support.

#### Looking back or fighting for the future

The young people described their future with ambivalence. One young person said, “But you know I think about the future, what’s gonna happen? What will I be? (17-year old male, P4)” Some described the future with feelings of worry and in some case hopelessness, others with feelings of great hope or the notion that the future was something worth fighting for. Even though these events were described with ambiguous feelings, it was clear to the young people that finishing school and having a job were important. One young person said, “When I think of the future…then I see people having to manage secondary school and get an education and then work” (18-year old male, P10). Finishing school or improving their grades was described as a challenge and something they had to work very hard to manage. Many described losing time due to unfortunate life events during childhood, which was described as stressful. Thinking about education and employment also caused them to reflect on their abilities to manage. Not everyone wanted to study and those that did not expressed that school was not for them, but this resulted in feelings of hopelessness as they saw education as the only route to employment.

Many described struggling to have what they called “a normal life.” This term included having something to do, like work or study, and earning money in a legal way. They described having their own family, being employed, and having friends as things that would make them feel good in the future. They also formulated future aspirations such as getting a higher degree, with the aim of working as a nurse, engineer, or police officer. The young people connected their future dream job with the desire to feel safe. Working as a police officer was described by one young person in this manner:

If you are the police you feel safe, you feel safe if you are a police officer. No one can get to you and yea… like my brother, my brother use to say that I’m a cop so nobody can do nothing, not hit me or anything or assault me or anything. So yea that people feel safe is important and if you yourself feel safe then you can do something for others… (17-year old male, P4)

A wish to settle down was expressed by the young people: just being where they were right now, not having to move again. One young person said “Life just stop changing. Future… (18-year old male, P10)”. The wish for life to be the same was also described with nostalgia. Some described life as good before but harder now and others took the position that life has been tough and cannot get any worse, it must get better. One young person described a wish to go back and relive childhood,

Then I want to move back to the village where I lived … that’s where I felt the very best… Yes, as I said, it was where I liked it the most. That’s simply it, I felt at home there, yea. So it’s something I’d like to do but it might not come true…. same house, same place, nothing different. (16-year old male, P2)

In order to feel good in their current and future daily life, the necessary ingredients included feeling safe, being with others, and not being alone. Words that were used to describe a good life included “peace and quiet” and “no war.” Overall, the theme *being forced to question what has been taken for granted* shows how the young people understood health as something changeable over time and space. The theme also illustrates how these life-changing experiences could have a positive or negative influence over young people’s sense of the future.

## Discussion

The aim of the study was to explore how young people in Sweden who neither work nor study perceive life experiences in relation to health and well-being. The findings consisted of three themes: *struggling with hardships in the absence of caring connections, feeling good when closely connected to others*, and *being forced to question what has been taken for granted*. These themes create a complex and diverse picture of experiences that promote or undermine health and well-being from the perspective of young people in Sweden who neither work nor study. The findings show how the participating young people were dealing with hardship and struggled for a sense of belonging. Within all themes a broad spectrum of experiences exists, which are described in depth in the sub-themes. Our comprehensive understanding of the themes and subthemes are that health, based on the young people’s narrated experiences could be understood as: something that is created in relation to others and in relation to the social and cultural context; as something dynamic and changeable; as the ability to adapt and respond to challenges; and finally as something existing on a collective as well as an individual level.

Health as something that is created in relation to others and in relation to the social and cultural context underscores how health is something more than a purely biological condition and the absence of disease or infirmity (WHO, 1946). Thus, this perspective illustrates the social dimensions of health and the importance of social relationships and networks to enhance the well-being of the individual (Hjelm, 2010; Wilkinson & Marmot, 2003). According to the findings, meaningful social relations and social support can counteract social exclusion and promote social inclusion. However, relationships to others can also create or strengthen feelings of exclusion. This is in line with Larsson et al. (2012); relationships with others can be a source of well-being as well as a cause of feelings of loneliness, otherness, and exclusion. Furthermore, in line with Pålsson (2012), we argue that inclusion and exclusion, which affect young people’s health and well-being, are not static positions. Rather, our findings show that there is a back-and-forth movement between feeling inside or outside a community. The experiences of inclusion and exclusion described by the young people can be found on different levels including the family, group of friends, community, and nation. However, social networks on different levels change over time and space, and so do experiences of health. In other words, health can be understood as something dynamic and changeable, which echoes Antonovsky’s (1987) approach to health as a continuum in which an individual in different situations is closer or further away from that which is perceived as good health.

Other key aspects of health in relation to this group of young people are opportunities to realize their life goals despite feelings of anxiety when thinking about the future. Health can therefore be seen as the ability to adapt and respond to challenges (Huber et al., 2011), which was reflected in the participants stories. They indicate willingness to and capability to start over by identifying resources and people helping them in the transition from hardship to a better life. Even when they expressed not knowing what it was like to feel well or not knowing what to do when dealing with hardship, they had the ability to express their needs, which according to the WHO (1986), can be seen as a necessary prerequisite to experiencing good health. We also argue that the view of health presented by Huber et al. (2011) as the ability to adapt and respond to challenges should be seen in relation to the social determinates of health described by Marmot (2009). In other words, the actual space in which an individual can take action depends on structural factors such as childhood living conditions, access to social support, and factors such as gender, class, and ethnicity.

However, we need to acknowledge that young people from war-torn environments coming to live in Sweden also experience hardships as well as positive health experiences commonly associated with childhood and adolescence similar to young people born and raised in Sweden. This study showed that young people growing up in Sweden experienced homelessness and rootlessness also found in the experiences of youth leaving their home countries for an uncertain future. Thus, although the young people in this study are a diverse group of individuals coming from different backgrounds (i.e., social class, ethnicity, and gender), they still share some common experiences related to feelings of inclusion and exclusion. In other words, it is important to take both individual and collective aspects of youths’ health in consideration. By such an approach, it is possible to counteract stereotypical views on youths’ health, as well as avoiding explaining hardships and positive experiences of health solely with regard to ethnical backgrounds.

Finally, the findings elucidate experiences among the young people including self-perceived discrimination or concerns about discrimination as well as a lack of trust in others and a lack of social support. Such experiences indicate a lack of participation, which should be taken into account because participation is highly prioritized in the Swedish public health goals (SOU, 2002). As was stated in the introduction, health in Swedish youth has become increasingly unequal (Public Health Agency of Sweden, 2013). Therefore, we argue in line with Povlsen et al. (2011) for a stronger focus on equity in health within health promotion in the Nordic context.

### Methodological reflections

The use of qualitative content analysis enabled exploration of the experiences of the group of young people who neither work nor study on a collective level, at the same time as we took the rich variations in their individual experiences in consideration. We therefore decided to choose themes that communicate the commonalities of the young people’s experiences and then formulated the sub-themes to widen the understanding of the great differences within the commonalities. With this study we show the variety of experiences in this group of young people and at the same time we acknowledge in line with Furlong (2006) and Nordenmark et al. (2015) the value of deepening the knowledge of the different subgroups within this heterogeneous group.

In addition, the use of a timeline as a task-based activity during the interview made it possible to explore in detail the meanings of significant events in young peoples´ lives, and to produce knowledge that can be of importance for professionals working with support activities for young people who are neither in school nor employed. Some participant had an easier time relating to and describing their life in relation to the timeline than others. As Johansson (2005) explains, describing life in a linear way can be seen as a cultural product taken for granted in western society but is not an approach that everyone can relate to.

Another methodological reflection concerned that the participant stated that they were stressed, tired, or had to go to other activities. This can, according to Mahon et al. (1996), be potential signs of lack of interest. The interviewer respected this and therefore two interviews were shorter than the rest. Furthermore, some of the participants initially expressed that they had not experienced any life events that gave them a sense of well-being. When a participant stated this, the first author asked if they wanted to describe some other significant life event. To maintain the focus on health promotion, the first author returned to the question “was there something during this period of life, during this hardship that made you feel better?” This probe led to stories, for example ones that described social support during hard times and the importance of relationship for the well-being of the young people.

Additionally, after the interview some participants commented that they enjoyed talking about their life from a life history perspective, that if “felt good to have gone through it all in that order” and that they now “understood what they have to do to feel better.” Moreover, they communicated that they were not used to talking about or reflecting on their health, however they really enjoyed it and found it meaningful. These comments suggest that the task-based activities gave the participants an opportunity to reflect and relate their life stories to experiences of health and well-being (cf. Conolly, 2008), which furthermore, coincides with the notion of empowerment described in the Ottawa Charter (WHO, 1986). Therefore, we argue in line with Larsson et al. (2012) that creating more opportunities and forums for young people to talk about and reflect over their health in relation to their life situation can support their health and well-being.

### Implications for practice

This study may contribute to an understanding of how young people in Sweden who neither work nor study perceive health in relation to their life experiences, which can be helpful for health promotion with this group of young people. First of all, the results reveal that this group of young people is a heterogeneous group and we suggest that if referring to them with the term NEET (Nordenmark et al., 2015) it is important to keep in mind the wide variety of hardships they need to concur. The results clearly show the importance of social support, and thus mentorship in school could be one way to strengthen the social networks of vulnerable young people in school (Schwartz, Rhodes, Spencer, & Grossman, 2013).

Furthermore, the participants expressed a wish to talk about their health, and this could be done within a mentorship as well as in a health dialogue at school. In previous studies, this has been shown to be beneficial for vulnerable young people (Borup & Holstein, 2004). In general, a stronger focus on health promotion in school could not only enhance the young people’s health but also improve their learning (Ahonen, 2010; Fröjd et al., 2008). In addition, the young people in this study expressed a need to be listened to and to be treated as individuals (in relation to social services and contact with adults). At the same time, the result illustrates some common experiences among the youth related to feelings of exclusion and inclusion.

In Sweden, the school’s ability to compensate for students’ background has worsened, and the parents’ level of education is becoming increasingly important for the students’ possibilities to succeed in school (Jobér, 2012, 2015). Therefore, initiatives with the ambition to empower young people must take their social and cultural background (often related to structural factors such as social class and ethnicity) in consideration and at the same time not letting such factors function as a social stigma regulating any expectations of the individual and her/his potential.

To accomplish this, Jobér (2012, 2015) has stressed that it is important to empower children from socially disadvantaged homes, i.e., by supporting toll-free school activities. Furthermore, it is important to regard talent not as an essential or static quality of the individual, but as something that can be created and encouraged with the right pedagogical tools. Thus, it is necessary to recognize the importance of social and cultural factors, but not regard them as determining for young people’s achievements.

Finally, the young people in this study expressed that there is no simple pre-made fixed solution, and they need to be involved in matters affecting their health and well-being—findings that concur with Head (2011). Involving young people makes them feel recognized for their competence and the participants in this study stated that this promotes their well-being. Furthermore, by giving young people an opportunity to participate, they can contribute with valuable knowledge about how to help them promote their own health and well-being. Our recommendation is thus to increase the amount of participation of young people who neither work nor study in health promotion efforts.

## References

[CIT0001] Ahonen A. (2010). *Psychosocial well-being of schoolchildren in the barents region. A comparison from the Northern parts of Norway, Sweden and Finland and Northwest Russia* (Doctoral thesis) University of Lapland, Rovaniemi, Finland.

[CIT0002] Antonovsky A. (1987). *Unraveling the mystery of health: How people manage stress and stay well*. San Francisco, CA: Jossey-Bass.

[CIT0003] Borup I., & Holstein B. E. (2004). Social class variations in schoolchildren’s self-reported outcome of the health dialogue with the school health nurse. *Scandinavian Journal of Caring Sciences*, 18, 343–12. 15598241 10.1111/j.1471-6712.2004.00302.x

[CIT0004] Conolly A. (2008). Challenges of generating qualitative data with socially excluded young people. *International Journal of Social Research Methodology*, 11(3), 201–214.

[CIT0005] Coyne I. (2010). Accessing children as research participants: Examining the role of gatekeepers. *Child: Care, Health and Development*, 36(4), 452–454. 10.1111/j.1365-2214.2009.01012.x 20642564

[CIT0006] Currie C. (Eds.) (2012). *Social determinants of health and well‐being among young people. Health Behaviour in School‐aged Children (HBSC) study: International report from the 2009/2010 survey* (Health Policy for Children and Adolescents, No. 6). Copenhagen: WHO Regional Office for Europe.

[CIT0007] Einberg E.-L., Lidell E., & Clausson E. K. (2015). Awareness of demands and unfairness and the importance of connectedness and security: Teenage girls’ lived experiences of their everyday lives. *International Journal of Qualitative Studies on Health and Well-Being*, 10, 27653. 26084273 10.3402/qhw.v10.27653 PMC4471215

[CIT0008] Fröjd S. A., Nissinen E. S., Pelkonen M. U. I., Marttunen M. J., Koivisto A.-M., & Kaltiala-Heino R. (2008). Depression and school performance in middle adolescent boys and girls. *Journal of Adolescence*, 31, 485–498. 17949806 10.1016/j.adolescence.2007.08.006

[CIT0009] Furlong A. (2006). Not a very NEET solution: Representing problematic labour market transitions among early school-leavers. *Work, Employment and Society*, 20(3), 553–569.

[CIT0010] Graneheim U. H., & Lundman B. (2004). Qualitative content analysis in nursing research: Concepts, procedures and measures to achieve trustworthiness. *Nurse Education Today*, 24(2), 105–112. 14769454 10.1016/j.nedt.2003.10.001

[CIT0011] Hagquist C. (2010). Discrepant trends in mental health complaints among younger and older adolescents in Sweden: An analysis of WHO Data 1985-2005. *Journal of Adolescent Health*, 46(3), 258–264. 20159503 10.1016/j.jadohealth.2009.07.003

[CIT0012] Hagquist C. (2011). Ökar den psykiska ohälsan bland ungdomar i Sverige? [Increases mental illness among young people in Sweden?]. *Socialmedicinsk Tidskrift*, 88(6), 474–485.

[CIT0013] Hammarstrom A., & Janlert U. (2002). Early unemployment can contribute to adult health problems: Results from a longitudinal study of school leavers. *Journal of Epidemiology Community Health*, 56(8), 624–630. 12118056 10.1136/jech.56.8.624 PMC1732218

[CIT0014] Head B. W. (2011). Why not ask them? Mapping and promoting youth participation. *Children and Youth Services Review*, 33, 541–547.

[CIT0015] Hjelm J. R. (2010). *The dimensions of health: Conceptual models*. Subury, MA: Jones and Bartlett Publishers.

[CIT0016] Huber M., Knottnerus J. A., Green L., van der Horst H., Jadad A. R., Kromhout D., … Smid H. (2011). How should we define health? *BMJ*, 2011(343), d4163. 10.1136/bmj.d4163 21791490

[CIT0017] Jobér A. (2012). *Social class in science class* (Doctoral thesis). Malmö University & Lund University, Sweden.

[CIT0018] Jobér A. (2015). *Social klass i skolan. Det kompensatoriska uppdraget* [Social class in school]. Stockholm: Natur och kultur.

[CIT0019] Johansson A. (2005). *Narrativ teori och metod* [Narrative theory and method]. Lund, Sweden: Studentlitteratur.

[CIT0020] Kostenius C. (2008). *Giving voice and space to children in health promotion* (Doctoral thesis). Luleå University of Technology, Luleå, Sweden.

[CIT0021] Kvale S., & Brinkmann S. (2009). *Den kvalitativa forskningsintervjun* [The qualitative research interview]. Lund, Sweden: Studentlitteratur.

[CIT0022] Larsson M., Johansson Sundler A., & Ekebergh M. (2012). The influence of living conditions on adolescent girls’ health. *International Journal of Qualitative Studies on Health and Well-Being*, 7. doi:10.3402/qhw.v7i0.19059 PMC3517721 23237626

[CIT0023] Mahon A., Glendinning C., Clarke K., & Craig G. (1996). Researching children: Methods and ethics. *Children & Society*, 10, 145‐154.

[CIT0024] Marmot M. (2005). Social determinants of health inequalities. *Lancet*, 365, 1099–1104. 15781105 10.1016/S0140-6736(05)71146-6

[CIT0025] Marmot M. (2009). Social determinants and adolescent health. *International Journal of Public Health*, 54, 125–127. 19639261 10.1007/s00038-009-5402-z

[CIT0026] Morrow V., & Richards M. (1996). The ethics of social research with children: An overview. *Children & Society*, 10, 90–105.

[CIT0027] Nardi B., Lucarellia C., Talamontia M., Arimateaa E., Fiorib V., & Moltedo-Perfettid A. (2015). NEETs versus EETs: An observational study in Italy on the framework of the HEALTH25 European project. *Research in Post-Compulsory Education*, 20(4), 377–399.

[CIT0028] National Board of Health (2009). *Folkhälsorapport* [Public Health Report]. Stockholm: Socialstyrelsen.

[CIT0029] Niknami S., & Schröder L. (2014). *Bakom siffrorna - unga som varken arbetade eller studerade 2000–2010* [Behind the numbers - young people neither working nor studying 2000-2010]. Stockholm: Myndigheten för ungdoms- och civilsamhällesfrågor.

[CIT0030] Nordenmark M., Gillander Gådin K., Selander J., Sjödin J., & Sellström E. (2015). Self-rated health among young Europeans not in employment, education or training with a focus on the conventionally unemployed and the disengaged. *Vulnerable Groups & Inclusion*. doi:10.3402/vgi.v6.25824

[CIT0031] Pålsson D. (2012). *“Jag är bara 15 år, men ibland känns det redan som att det är kört”. Ungas röster om socialt utanförskap i Sverige* [“I’m only 15 years, but sometimes it feels like it’s already too late” Young people’s voices on social exclusion in Sweden]. Sweden: UNICEF.

[CIT0032] Polit D. F., & Beck C. T. (2004). *Nursing research. Principles and methods* (7nd ed.). Philadelphia: Lippincott, Williams & Wilkins.

[CIT0033] Povlsen L., Borup I. K., & Fosse E. (2011). The concept of “equity” in health-promotion articles by Nordic authors – A matter of some confusion and misconception. *Scandinavian Journal of Public Health*, 39(6), 50–56. 10.1177/1403494810376852 21382848

[CIT0034] Public Health Agency of Sweden (2013). *Barn och unga 2013: Utvecklingen av faktorer som påverkar hälsan och genomförda åtgärder* [Children and adolescents 2013 developments in health determinants and implemented measures]. Östersund, Sweden: Statens Folkhälsoinstitut.

[CIT0035] Randell E., Jerdén L., Öhman A., & Flacking R. (2016). What is health and what is important for its achievement? A qualitative study on adolescent boys’ perceptions and experiences of health. *The Open Nursing Journal*, 10, 26–35. 27347252 10.2174/1874434601610010026 PMC4894944

[CIT0036] Rutberg S., Kostenius C., & Öhrling K. (2013). Professional tools and a personal touch – Experiences of physical therapy of persons with migraine. *Disability and Rehabilitation*, 35(19), 1614–1621. 23311671 10.3109/09638288.2012.748838 PMC3786518

[CIT0037] Safipour J., Higginbottomb G., Mesfin K. T., & Emamid A. (2012). Migration status and self-reported health among high school students in Stockholm: A cross-sectional study. *Vulnerable Children and Youth Studies*, 7(2), 149–163.

[CIT0038] Sand A., Emaus N., & Lian O. (2015). Overweight and obesity in young adult women: A matter of health or appearance? The Tromso study: Fit futures. *International Journal of Qualitative Studies on Health and Well-Being*, 10. doi:10.3402/qhw.v10.29026 PMC4580710 26400463

[CIT0039] Schwartz S. E. O., Rhodes J. E., Spencer R., & Grossman J. B. (2013). Youth initiated mentoring: Investigating a new approach to working with vulnerable adolescents. *American Journal of Community Psychology*, 52, 155–169. 23780477 10.1007/s10464-013-9585-3

[CIT0040] SFS 2003:460. *Lag om etikprövning av forskning som avser människor* [Law on ethical approval of research on human beings]. Retrieved 617, 2015, from https://www.notisum.se/rnp/SLS/LAG/20030460.HTM.

[CIT0041] Sofaer S. (1999). Qualitative methods: What are they and why use them? *Health Services Research*, 34(5 II), 1101–1118. 10591275 PMC1089055

[CIT0042] SOU (2002). *Mål för folkhälsan. (2002/03:35)* [Public health objectives]. Stockholm: Socialdepartmentet.

[CIT0043] SOU (2013). *Ungdomar utanför gymnasieskolan – ett förtydligat ansvar för stat och kommun (2013:13)* [Young people outside the secondary school - an explicit responsibility for state and local government]. Stockholm, Sweden: Fritzes Offentliga Publikationer.

[CIT0044] Stengård E., & Appelqvist-Schmidlechner K. (2010). *Mental health promotion in young people – An investment for the future*. Copenhagen: WHO Regional Office for Europe.

[CIT0045] UNICEF (2011). *The state of the world’s children. Adolescence: An age of opportunity*. New York: Author.

[CIT0046] Valentine G., Butler R., & Skelton T. (2005). The ethical and methodological complexities of doing research with ’vulnerable’ young people In Sheehy K. (Ed.), *Ethics and research in inclusive education: Values into practice*. London: Routledge Falmer.

[CIT0047] WHO (1946). *Official records of World Health Organization, no 2*. Retrieved 1117, 2016, from http://who.int/about/definition/en/

[CIT0048] WHO (1986). *Ottawa charter for health promotion*. Genève: World Health Organization.

[CIT0049] Wilkinson R., & Marmot M. (2003). *Social determinants of health: The solid facts* (2nd ed.). Copenhagen: WHO Regional Office for Europe.

[CIT0050] Woodgate R. L., & Leach J. (2010). Youth perspectives on the determinants of health. *Qualitative Health Research*, 20(9), 1173–1182. 20442342 10.1177/1049732310370213

